# Preoperative Imaging and Online Photo Galleries: The #Key to Surgical Commitment

**DOI:** 10.1055/s-0041-1739117

**Published:** 2021-12-15

**Authors:** Victoria B. Givens, Stephen W. Perkins

**Affiliations:** 1 Givens Facial Plastic Surgery - Facial Plastic Surgery, Austin, Texas; 2 Meridian Plastic Surgery Center, Facial Plastic Surgery, Indianapolis, Indiana

**Keywords:** preoperative imaging, facial rejuvenation, rhinoplasty, social media, surgical commitment

## Abstract

**Importance** Preoperative imaging provides an advantageous balance by helping patients to effectively communicate their aesthetic desires while allowing surgeons to establish realistic expectations of surgical outcomes.

**Objective** To determine the role of preoperative imaging and the importance of online-based photo galleries in influencing a patient's decision to pursue cosmetic facial plastic surgery.

**Design, Setting, and Participants** A retrospective study was conducted on 100 patients who underwent preoperative imaging prior to undergoing aesthetic facial plastic surgery from July 2019 to May 2020. An in-office physician-led clinical consultation followed by a preoperative imaging session was performed on each patient prior to surgical intervention. A 6-question survey was provided once to all patients between their 3- and 12-month postoperative time periods.

**Main Outcomes and Measures** The importance of preoperative imaging and the influence of physician website and social media photo galleries regarding surgical decision-making was evaluated.

**Results** A total of 100 participants (female [90; 90%]) and mean age 52.6 (range, 18–77) years were included. Nearly 60% of patients underwent facial rejuvenation procedures. All reported that preoperative in-office physician consultation in combination with the use of preoperative imaging were helpful in facilitating a commitment to surgical intervention. Sixty-nine (69%) patients endorsed the use of both the frontal and lateral imaging views, while 30 (30%) deemed a single angle to be superior. Seventy (70%) participants utilized online-based “before & after” photo galleries in the form of physician websites and/or social media platforms to assist in their decision to undergo surgical intervention.

**Conclusions and Relevance** The combination of in-office physician consultation, preoperative imaging, and availability of website and/or social media photo galleries plays a key role in a patient's decision to pursue cosmetic surgery. Thus, implementation of all facets should become an integral part of any facial plastic surgeon's aesthetic practice.

## Introduction

Regarding the aesthetic focus of facial plastic surgery patients, appearance is of top priority. Upholding patient expectation via production of a “rejuvenated yet natural” result is a delicate balancing act. The mere perception in degree of change to one's facial appearance along with the realization that too far a swing of the metaphorical pendulum in one direction brings out an uncanny uncertainty in patients, which can easily translate into deferment of surgical intervention, even after consultation with the most articulate and seasoned surgeon. This is where preoperative imaging both defines and solidifies its hold firmly in the facial plastic surgeon's armamentarium.

Advancements in technology over the years have allowed surgeons to display for patients not only what they currently look like but also the degree to which an aesthetic procedure can remedy a displeasing aberration, all in real-time. Preoperative imaging has the ability to visually demonstrate outcomes of potential procedures in patients who are either not sure which procedures (s) they want to commit to or when deciphering if the benefits of one procedure outweighs those of another. 1 2 3 4 5 In essence, preoperative imaging actually provides patients with realistic possibilities, all the while building trust in the physician-patient relationship, so that a commitment to pursue surgical intervention can be made confidently. 1 2 3 4 5

Our study's objective was to determine the role of preoperative in-office consultation imaging and the importance of online-based photo galleries in influencing a patient's decision to pursue cosmetic facial plastic surgery.

## Methods

**Fig. 1:**
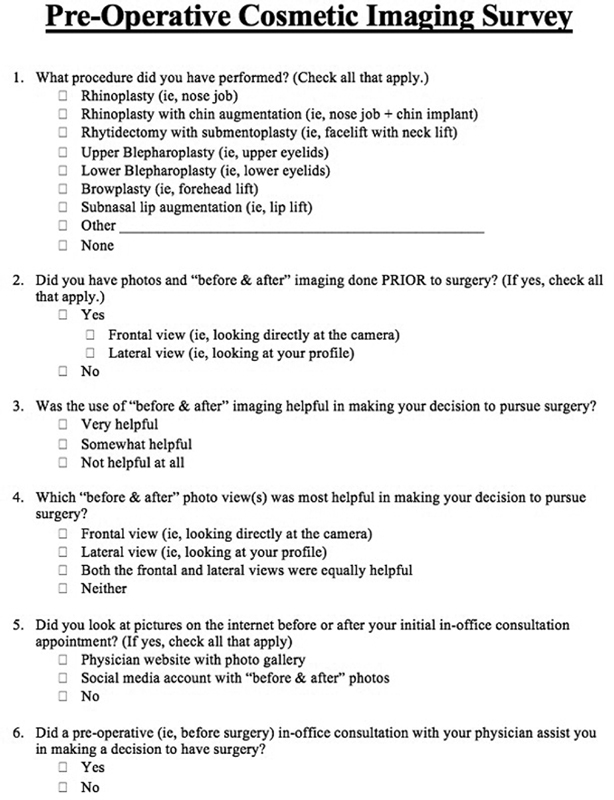
The 6-question survey provided to all participants once between their 3- and 12-month postoperative time periods.

A retrospective study was conducted on patients at our institution who underwent preoperative imaging prior to undergoing cosmetic facial plastic surgery from July 2019 to May 2020. Upon initial clinical consultation, each patient was evaluated by the senior author (S.W.P.). Evaluation began with a thorough history and physical examination and focused assessment of patient aesthetic goals. Complete evaluation of anatomic features including areas with structural aging, asymmetry, and/or deformity was performed followed by manual demonstration of surgically achievable results to the patient using a three-way mirror for full effect. Standardized photographs based on patient aesthetic area of concern were then obtained by our institution's photographer and archived into our imaging software system (United Imaging, Hillsborough, North Carolina). Digital alteration of these images in the anteroposterior (AP) and left profile views was then performed by our institution's imaging consultation specialist. Preoperative photographs and imaging were reviewed and approved by the patient, senior author, and imaging consultation specialist at the same time. Each patient's preoperative photographs were displayed in the operating room for use in the intraoperative period. At the postoperative 3-, 6-, and 12-month intervals, follow-up photographs were obtained. A six-question survey assessing the importance of preoperative imaging and the influence of physician website and social media photo galleries regarding surgical decision-making was provided once to all patients between their 3- and 12-month postoperative time periods ( Fig. 1 ). A total of 100 surveys was collected for review.

Statistical analysis was not pertinent to this study. Approval was obtained from the American Academy of Facial Plastic & Reconstructive Surgery's Fellowship Research Review Subcommittee.

## Results

A total of 100 participants successfully completed surveys between July 2019 and May 2020. The majority were female (90 [90%]), and the mean age was 52.6 (range, 18–77) years ( Table 1 ). Most patients underwent facial rejuvenation procedures (FRPs), which included facelift (FL), blepharoplasty, browplasty, and/or subnasal lip augmentation for the purposes of this study. Fifty-eight (58%) patients underwent facial rejuvenation procedures alone, 40 (40%) underwent rhinoplasty ± chin augmentation as well as FRPs, and 2 (2%) underwent chin augmentation with FRPs.

**Table 1:** Patient demographics and outcomes

Patient Demographics ( *n* = 100)	No. (%)
Gender (female)	90 (90%)
Age (range in years)	52.6 (18–77)
**“Helpfulness” of imaging**	
Very	85 (85%)
Somewhat	15 (15%)
Not	0
**Preferred imaging view**	
Frontal	6 (6%)
Lateral (Profile)	24 (24%)
Both	69 (69%)
Neither	1 (1%)
**Use online photo galleries**	
Physician website	45 (45%)
Social media platform (s)	4 (4%)
Both	21 (21%)
Neither	30 (30%)

Of the 58 facial rejuvenation procedures, FL alone was performed on 14 (24.1%) patients. Thirty-two (55.2%) patients underwent FL in addition to blepharoplasty, browplasty, and/or subnasal lip augmentation (15 FL + blepharoplasty, 4 FL + browplasty, 13 FL ± blepharoplasty/browplasty/lip augmentation). The remaining 12 (20.7%) patients underwent blepharoplasty and/or browplasty.

Of the 40 rhinoplasty procedures ± additional surgical intervention, rhinoplasty alone was performed on 25 (62.5%) patients. Fifteen (37.5%) patients underwent rhinoplasty along with chin augmentation and/or facial rejuvenation surgery (8 rhinoplasty + FRPs, 5 rhinoplasty + chin augmentation, 2 rhinoplasty/chin augmentation + FRPs). In addition to the above patients, two participants underwent chin augmentation and FRPs (2 FL + blepharoplasty).

All participants reported that their preoperative in-office physician consultation in combination with the use of preoperative imaging were helpful in facilitating a decision to pursue surgery. The majority (85 [85%]) found the preoperative imaging session “very helpful” with the remainder (15 [15%]) considering it “somewhat helpful” ( Table 1 ). No patients found preoperative imaging “unhelpful.”

Survey response demonstrated that the majority (69 [69%]) of patients found both the frontal and lateral preoperative imaging views to be most helpful in making the decision to pursue surgical intervention ( Table 1 ). Thirty (30%) patients found either the frontal view (6 [20%]) or the lateral view (24 [80%]) alone to be most helpful. One patient found neither imaging view significantly helpful in making the decision to pursue surgery.

Of the 69 patients who found both views to be most helpful for decision making, 41 (59.4%) chose to pursue FRPs (8 FL only, 33 FL ± blepharoplasty/browplasty/lip augmentation). Twenty patients (28.9%) underwent rhinoplasty ± chin augmentation (18 rhinoplasty only, 2 rhinoplasty + chin augmentation). Six patients (8.7%) chose to pursue a combination of rhinoplasty ± chin augmentation in addition to FRPs (4 rhinoplasty + FL, 2 rhinoplasty ± chin augmentation + FL/blepharoplasty). Two patients (2.9%) underwent chin augmentation with FL/blepharoplasty.

While 30 patients found only one view to be most helpful with regard to surgical commitment, the majority (24 [80%]) found the lateral view superior. Of those who preferred the profile view, fourteen (58.3%) patients underwent FRPs only (13 FL ± blepharoplasty/browplasty/lip augmentation, 1 browplasty only), nine (37.5%) underwent rhinoplasty ± chin augmentation (6 rhinoplasty only, 3 rhinoplasty ± chin augmentation), and 1 (4.2%) underwent rhinoplasty + FL. Of the 6 patients who found the frontal view alone to be most helpful in their decision to pursue surgery, three underwent rhinoplasty only, one underwent FL only, one underwent rhinoplasty + FL, and one underwent browplasty only.

Whether or not online-based photo gallery review was performed by each patient was studied. While the majority (70 [70%]) of patients utilized an Internet-based photo gallery review of surgical procedure outcomes, 30 (30%) patients did not ( Table 1 ). Of the 70 patients who did employ web-based research, 45 (64.3%) used the consulting physician's website photo gallery, four (5.7%) examined social media platforms with available “before & after” photos, and 21 (30%) engaged both.

Of the 21 patients who used both physician website and social media photo galleries, the mean age was 47.1 (range, 30–68) years and selected procedures included rhinoplasty ± chin augmentation (8 [38.1%]), rhinoplasty with FRPs (4 [19%]), and FRPs only (9 [42.9%]). Of the 45 patients who used the physician's website photo gallery only, the mean age was 49.9 (range, 18–71) years and selected procedures included rhinoplasty ± chin augmentation (17 [37.8%]), rhinoplasty with FRPs (3 [6.7%]), and FRPs only (25 [55.6%]). Of the four patients who utilized social media platforms, the mean age was 55.8 (range, 44–68) years and selected procedures included rhinoplasty ± chin augmentation (2 [50%]), rhinoplasty with FRPs (1 [25%]), and FRPs only (1 [25%]). Of the 30 patients who did not use any form of online-based photo gallery review, the mean age was 59.9 (range, 19–75) years and selected procedures included rhinoplasty ± chin augmentation (3 [10%]), rhinoplasty with FRPs (4 [13.3%]), and FRPs only (23 [76.7%]).

## Discussion

The infusion of medical photography and preoperative imaging captures the future visual representation of the human body in real-time. To the facial plastic surgeon consulting with a new patient, it is a vital communication tool. In essence, preoperative imaging provides a necessary balance by helping patients to effectively communicate their aesthetic desires while allowing surgeons to establish realistic expectations of surgical outcomes.

Our study measured a significant trend in favor of the use of preoperative in-office consultation imaging prior to obtaining a patient's surgical commitment. Of the 100 surveyed (58% facial rejuvenation, 40% rhinoplasty ± chin augmentation, 2% chin augmentation without rhinoplasty), all participants agreed that implementation of preoperative imaging was helpful in influencing their decision to pursue surgical intervention with 85 (85%) patients finding it “very helpful.” Although deemed “somewhat” helpful according to survey results, the use of preoperative imaging in the remaining 15 (15%) participants did play a crucial role in their decision to pursue surgical intervention. For these patients, the success of preoperative in-office consultation with their surgeon along with a well-established physician-patient relationship was tantamount. In effect, the patient was already committed to a surgical decision whether or not preoperative imaging was available for review.

All patients underwent preoperative imaging, which included both a frontal and lateral view for comparison, no matter the surgical procedure being considered. Nearly 70% of participants found that review of both angles was equally helpful in their decision to pursue surgical intervention. FRPs comprised the majority of cases at 59% with the remaining 41% undergoing either rhinoplasty ± chin augmentation (29%, rhinoplasty + FRPs [9%], or chin augmentation + FRPs [3%]). This reaffirms that while each view alone plays a significant role in patient understanding of individual anatomy and realistic surgical outcome, together they allow for a patient to fully commit to surgical intervention if there was any doubt prior to review.

Thirty (30%) participants found only one preoperative imaging view to be superior, and a single patient found neither view especially helpful. The latter patient was already resolved to have surgery, whether or not preoperative imaging was performed. Of those who selected for a preferential angle, the majority (24 [80%]) indicated that the lateral preoperative imaging view was superior. FRPs were most commonly performed at 58.3% with the remaining 41.7% of patients undergoing rhinoplasty ± chin augmentation (37.5%) or rhinoplasty + FRPs (4.1%). This portends that a profile view of a neckline and/or nose captures the main anatomical aberration that a patient desires to be surgically corrected. In comparison, a total of 6 patients (3 rhinoplasty only, 1 FL only, 1 rhinoplasty + FL, 1 browplasty only) considered the frontal view alone to be most helpful in their decision to pursue surgery. While both uncommon and unique for a patient undergoing a FL and/or rhinoplasty to find the lateral pre-operative imaging view not useful, it is not unprecedented. In these patients, it is perhaps a particular angle of the nose or laxity of the face/neck demonstrated best on frontal view which is of utmost concern. After all, the frontal view is what we as humans actually appreciate in the mirror on a daily basis and not the lateral view.

Our study also measured an overall positive trend in favor of the use of Internet-based patient research via physician website and/or social media platform “before & after” surgical photo galleries. Seventy percent of surveyed participants utilized online visual review of pre- and postoperative outcomes, while the remaining 30% did not use any supplementary materials in addition to in-office physician consultation and preoperative imaging. This paramount disparity is most likely attributable to the overall difference in age-related importance of technological application between the two groups. The mean age was 50.3 (range, 18–71) and 59.9 (range, 19–75) years between those who performed online research and those who did not, respectively. While a mere decade of difference in age may not at first appear to hold significant weight, it most certainly does from a technological advancement standpoint.

The world wide web first became publicly available 1990, Facebook launched in 2004, and Instagram was unveiled in 2010. This would make those born in 1960 (∼ 59 years old) already 30 years old before the Internet even launched. In addition, this same age group was already 44 and 50 years old before the release of Facebook and Instagram, two of the most prominent social media platforms to host visual “before and after” surgical outcomes today, respectively. Thus, it is no surprise that those nearly 60 years old today do not have a strong vested interest in online-based research via physician website and/or social media account “before & after” surgical photo galleries.

When comparing the 49 (49%) participants who utilized only one form of online gallery review, the use of physician website (45 [91.8%]) “before & after” photos was superior to social media-based platforms (4 [8.2%]). This largely stems from a combination of the cosmetic patient's desire to review their potential surgeon's work and build trust in their physician. In addition, the prevalent use of social media platforms centers on the culture of the region, the “focus and teachings” of prominent social media influencers in the area, and the importance of technological advancement in the community.

Our study demonstrated that participants who underwent FL ± other surgical intervention tended to use less or no online-based photo gallery review, while who underwent rhinoplasty ± chin augmentation were much more likely to utilize Internet-based photographic outcomes. The mean ages were 58.5 (range, 32–77) years for FL and 42.9 (range, 18–77) years for rhinoplasty ± chin augmentation. There were no major differences in the use of online-based research for patients who underwent blepharoplasty, browplasty, and/or lip augmentation when selected for separately. Performance of FRPs such as FL have a relatively linear correlation with respect to increasing age. This reiterates the observation our study made previously in that less online review of photos was performed by patients of older age groups when compared with the younger age group undergoing nasal reconstruction.

## Strengths and Limitations

This study had several strengths and limitations. Our study utilized the same surgeon (S.W.P.), photographer, and imaging team for every patient. The fixed cohesiveness of a team allows for not only a better dynamic with patients but also prevention of subtle changes that may alter perception and surveyed result outcomes. Thus, preoperative consultation and imaging review with the same physician, photographer, and imaging specialist is vital to patient-based surgical decision making.

Patients were only included in the study if they were able to complete the preoperative imaging survey between the 3- and 12-month postoperative time periods. These parameters were chosen to prevent patients from filling out a survey too early in their recovery process and/or too far outside of the timeline one would accurately remember the therapeutic usefulness of preoperative imaging in relation to the surgical decision-making process. However, postsurgical results continue to change even after a year from the time the original surgical intervention was performed. In addition, a 3-month postoperative result, while devoid of the initial acute postoperative swelling and ecchymosis, is still premature.

While the study observed that as age increases there is a linear correlation with performance of FRPs and a lack of patient-based Internet utilization, this may not always be the case, depending on the region. Cultural preference, social media influencer reach, technological awareness and adaptability, and personal aesthetic goals at any age are variable and widely dispersed. Expansion of the study to include more geographical regions would provide increased study power and a better understanding of how surgical procedures and the use of online-based photo galleries correlate with patient age.

Conversion rates were not obtained. The objective of the study was to ascertain the value of preoperative imaging and online photo galleries in patients who did schedule surgery rather than determine if these methods enhanced conversion rates. The overwhelming majority found these methods definitively valuable, and as such, the study's results demonstrated that the routine use preoperative imaging and photo galleries to be a worthwhile addition to one's practice. Conversion rates were not obtained, as these are extremely difficult to measure for many reasons, including but not limited to patient finances, personal schedules, family and/or personal emergencies, and individual medical contraindications to surgical intervention.

The study was limited to the senior author's practice patients' experience. Expanding a study of this kind to other aesthetic surgical practices would provide increased data, although extremely variable and nonstandardized. Including outside practices and patients would not significantly change the validity, purpose, and/or power of the study. In addition, direct comparison of the senior author's methods to those of other physicians was not necessary.

## Conclusion

When combined with in-office physician consultation, an individualized preoperative imaging session plays a key role in a patient's decision to pursue aesthetic surgery. In addition, the presence of online “before & after” photo galleries is often indispensable in facilitating a patient's decision to not only obtain a consultation appointment but also commit to surgical intervention. Thus, the implementation of preoperative in-office consultation imaging and availability of website and/or social media photo galleries should become an integral part of any facial plastic surgeon's aesthetic practice.
